# Alpha-1 antitrypsin deficiency and risk of lung cancer in never-smokers: a multicentre case–control study

**DOI:** 10.1186/s12885-022-09190-3

**Published:** 2022-01-19

**Authors:** Ramón Antonio Tubío-Pérez, María Torres-Durán, María Esmeralda García-Rodríguez, Cristina Candal-Pedreira, Julia Rey-Brandariz, Mónica Pérez-Ríos, Juan Barros-Dios, Alberto Fernández-Villar, Alberto Ruano-Raviña

**Affiliations:** 1 Pulmonary Department, Hospital Álvaro Cunqueiro, AS Vigo; NeumoVigo I+I Research Group, Galicia Sur Health Research Institute (IISGS), Galicia, Spain; 2 Department of Preventive Medicina and Public Health, University of Santiago de Compostela, Santiago, Spain; 3 Department of Preventive Medicina and Public Health, University of Santiago de Compostela, Spain CIBER de Epidemiología Y Salud Pública, CIBERESP, Santiago, Spain

**Keywords:** Alpha-1 antitrypsin, lung cancer, never-smokers

## Abstract

**Background:**

Lung cancer (LC) is the most commonly diagnosed cancer and the leading cause of cancer-related death in both sexes worldwide. Although the principal risk factor in the western world is tobacco smoking, genetic factors, including alpha-1 antitrypsin deficiency (AATD), have been associated with increased risk. This study is the continuation of an earlier one published by the same group in 2015, aimed at analysing risk of LC in never-smokers, associated with carriers of the AATD genotype.

**Methods:**

A multicentre case–control study was conducted in Spain across the period January 2011 to August 2019. Cases were non-smokers diagnosed with LC, and controls were composed of never-smoking individuals undergoing major non-cancer-related surgery. Data were collected on epidemiological characteristics, exposure to environmental tobacco smoke (ETS), residential radon levels, and alpha-1 antitrypsin (AAT) genotype.

**Results:**

The study included 457 cases (42%) and 631 controls (58%), with a predominance of women (72,8%). The most frequent histological type was adenocarcinoma (77.5%), followed by squamous cell carcinoma (7.7%). No association of risk of LC was found with the status of AATD genotype carrier, both overall and broken down by age, sex, or exposure to ETS.

**Conclusions:**

No risk association was found between being a carrier of an AAT deficiency genotype and LC among never-smokers. In order to establish the existence of an association, we consider it important to expand the studies in never smokers in different geographical areas as well as to include patients with previous chronic lung diseases to assess if it influences the risk.

## Background

Lung cancer (LC) is the most commonly diagnosed cancer and the leading cause of cancer-related death in both sexes worldwide [1, 2]. Currently it has an estimated 5-year survival rate of 13–20% [3]. Tobacco smoking is its principal risk factor, followed by exposure to radon, which is the second leading LC risk factor globally, and the leading LC risk factor among never-smokers [4].

LC among never-smokers has increased in incidence, accounting for 15% of all lung cancers in the USA in recent years [5]. It displays characteristics that distinguish it from LC in smokers, including a number of genes [6] and their protein products, which could act at different levels in the process of carcinogenesis and tumour progression. Among these is alpha-1 antitrypsin (AAT) [7, 8], which is a glycoprotein synthesized primarily by hepatocytes (80%) and, to a lesser extent, by other cells such as monocytes, macrophages or neoplastic cells among others [9]. It is encoded by the SERPINA1 gene, located in the long arm of chromosome 14 [10], and is transmitted by autosomal co-dominant Mendelian inheritance [11]. The PiMM genotype (Pi: protease inhibitor) is considered normal in structure and function [12], and is present in 85–90% of the Caucasian population [13]. Over 125 different allelic variants of this gene have been described: among these, the most frequent deficiency alleles are the PiS and PiZ alleles, with a frequency in Caucasians of 3–10% and 1–3%, and plasma antiprotease activity of around 60% and 20% of normal values, respectively [14]. The principal action of AAT is inhibition of serine protease, with neutrophil elastase being the most important at a tissular level [15]. Other properties described are anti-inflammatory, antimicrobial and immunomodulatory activity [16, 17], which has led to establish the possible involvement of AAT in the development and progression of different types of neoplasms [18], including LC [7, 19]. The organ most commonly affected by AATD in adults is the lung, through the development of emphysema, especially among individuals with exposure to tobacco smoke [20].

Some studies have analysed the possible relationship between AATD and lung cancer, with uneven results [21, 22]. A recent systematic review [23], which included 6 studies and a total of more than 4,000 patients, suggests that AATD could increase the risk of LC, and adenocarcinoma and squamous cell carcinoma in particular. This risk would increase with exposure to tobacco smoke and in individuals with diagnosis of COPD. Yet, there is only one study conducted on never-smokers which indicates an increased risk of LC associated with homozygous S-allele carriers (OR: 4.64 95% 95% confidence interval (CI) = 1.08–19.92). This risk is higher in women (OR: 7.58: 95% CI 1.40–40.87) and in subjects with exposure to environmental tobacco smoke (ETS) (OR: 12.10; 95% CI 1.18–123.77). This same study, undertaken by our group, is the only study to have taken exposure to residential radon into account [24].

The main aim of the present study was to analyse whether the fact of being a carrier of an AAT deficiency genotype increases the risk of LC among never-smokers. The secondary objectives were to analyse whether there might be differences in the result, on stratifying by age, sex and exposure to ETS.

We present the following article in accordance with the STROBE reporting checklist.

## Methods

We designed a multicentre hospital-based case–control study at 7 hospitals situated in north-east Spain (6 in Galicia and 1 in Asturias). The study participants were recruited across the period January 2011 to August 2019: cases were never-smoker patients histologically diagnosed with LC at the participating hospitals, and controls were never-smokers undergoing non-cancer-related surgery, most commonly major ambulatory surgery. Controls were enrolled using frequency sampling by sex and age with respect to cases. There were no restrictions in terms of sex, and all participants were over the age of 35 years. A never-smoker was defined as someone who had smoked < 100 cigarettes per lifetime or < 1 cigarette/day during a maximum of 6 months.

At the time of their inclusion in the study, all participants answered a survey, which included questions about the characteristics of their daily lives, with special stress on exposure to ETS. Exposure to ETS was defined as having cohabited with a smoker for a minimum of 20 years. Participants were asked about their personal relationship with the smoker, i.e., years of cohabitation and number of cigarettes/day smoked by the coinhabitant. An Alpha-track radon detector was delivered to all participants and placed in their bedrooms for the purpose of taking readings for a minimum period of 3 months. All detectors were read at and the results were then obtained from the Galicia Radon Laboratory (www.radon.gal), a facility certified by the Spanish National Accreditation Body (*Entidad de Acreditación Nacional*) for taking these types of measurements. All methods were carried out in accordance with relevant guidelines and regulations. [11] The study protocol was approved by the Galician Ethics Committee (2010/295).

### Laboratory analysis

A specimen of 3 ml of whole blood was taken from all the study subjects for determination of genetic polymorphisms, including AAT deficiency alleles S and Z for genotyping. All samples were analysed by the National Genotyping Centre (*Centro Nacional de Genotipado*) at the University of Santiago de Compostela using the MassARRAY iPLEX GOLD SNP genotyping system (Sequenom Inc., San Diego, CA) in accordance with the manufacturer’s instructions. This method has been described by Buetow et al. [25].

### Statistical analysis

Firstly, we conducted a bivariate analysis describing the characteristics of age, sex, educational level, exposure to ETS, residential radon concentrations, and distribution of the S or Z mutations of AAT with respect participants’ case or control status. A logistic regression was then performed, in which the dependent variable was case or control status, and the independent variable was being a homozygous or heterozygous S- or Z-allele carrier. The same analysis was performed by reference to age of over or under 65 years, and in women and men. Lastly, we analysed whether the risk of LC for patients with AAT deficiency alleles was different according to their exposure to ETS. Results were expressed as Odds Ratios with 95% CI and adjusted for age, sex and exposure to ETS. All analyses were performed using the SPSS computer software package version 20.

## Results

The study covered 457 cases and 631 controls. The characteristics of the participants are shown in Table 1: the median age of cases was slightly higher than that of controls, with a clear predominance of women (79.6%); 39.6% of cases and 31.7% of controls had residential radon concentrations ≥ 200 Bq/m^3^; 41.1% of cases had experienced exposure to ETS in the home during the preceding 20 years; 77% of cases were adenocarcinomas; 19.9% of cases and 26.6% of controls were carriers heterozygous for the S allele versus 2% of cases and 1.6% of controls who were homozygous for the S allele. In the case of the Z allele, 3.7% of cases and 4% of controls were heterozygous carriers, with no subject being a homozygous carrier.

**Table 1:** Characteristics of individuals included in the study

Variable	Cases, n (%)	Controls, n (%)
N	457 (42)	631 (58)
Median age (P25—P75)	70 (61–78)	66 (58–75)
Gender		
Female	364 (79.6)	429 (68.0)
Male	93 (20.4)	202 (32.0)
Educational level		
No formal education	156 (34.1)	177 (28.1)
Primary education	210 (46.0)	293 (46.4)
Secondary education	44 (9.6)	86 (13.6)
University education	47 (10.3)	75 (11.9)
Exposure to residential radon (Bq/m^3^)		
< 200	276 (60.4)	431 (68.3)
≥ 200	181 (39.6)	200 (31.7)
Exposure to environmental tobacco smoke		
Yes	193 (42.2)	259 (41.0)
No	264 (57.8)	372 (59.0)
Histological type		
Adenocarcinoma	356 (77.9)	
Squamous cell carcinoma	35 (7.7)	
Small-cell carcinoma	28 (6.1)	
Large-cell carcinoma	11 (2.4)	
Other histological types	27 (5.9)	
AAT mutations		
PI*S		
Not present	357 (78.1)	453 (71.8)
Heterozygous	91 (19.9)	168 (26.6)
Homozygous	9 (2.0)	10 (1.6)
PI*Z		
Not present	440 (96.3)	606 (96.0)
Heterozygous	17 (3.7)	25 (4.0)
Homozygous	0	0

*ETS* environmental tobacco smoke, *AAT* alpha-1 antitryps

Table 2 shows the analysis of risk of LC associated with different genotypes, exposure to ETS, and residential radon levels. No significant increase was observed in risk of LC with any of the genotypes analysed, and risk among homozygous subjects was no higher than among heterozygous subjects. Similarly, no increased risk of LC was found to be associated with exposure to ETS or residential radon concentrations ≥ 200 Bq/m^3^.

**Table 2:** AAT genotype and adjusted risk of LC

Variable	Cases, n (%)	Controls, n(%)	Adjusted OR^a^ (95% CI)	Adjusted OR^b^ (95% CI)
AAT	457 (42)	631 (58)		
MM	279 (73.0)	337 (66.7)	1 (-)	1 (-)
MS	80 (20.9)	137 (27.1)	0.67 (0.49–0.91)	0.66 (0.48–0.91)
MZ	10 (2.6)	18 (3.6)	0.79 (0.37–1.6)	0.64 (0.28–1.4)
SZ	4 (1.0)	4 (0.8)	1.09 (0.25–4.69)	0.9 (0.21–3.91)
SS	9 (2.4)	9 (1.8)	1.09 (0.42 -2.78)	1.1 (0.42–2.89)
ETS				
No	226 (59.2)	302 (59.8)	-	1 (-)
Yes	156 (40.8)	203 (40.2)	-	0.88 (0.66–1.17)
Radon exposure				
< 200 Bq/m3	220 (57.6)	315 (62.4)	-	1 (-)
> 200 Bq/m3	162 (42.4)	190 (37.6)	-	1.24 (0.94 -1.64)

^a^Adjusted for age and sex

^b^Adjusted for age, sex, and exposure to ETS and residential radon

*ETS* environmental tobacco smoke, *AAT* alpha-1 antitrypsin

Table 3 shows the characteristics of cases who were homozygous carriers of the SS genotype. These were 9 patients having a median age of 68 years (62–85), 88% women. Presence of emphysema in LC was found in none of the cases. The mean pulmonary function values were as follows: FEV1 (forced expiratory volume in the first second) 1.694 ml; FEV1 105.4%; and DLCO (diffusing capacity of the lung for carbon monoxide) 81%. The most frequent histology was adenocarcinoma (55%), followed by squamous cell carcinoma (45%).

**Table 3:** Characteristics of lung cancer cases among carriers of the PI*SS genotype

Cases (n)	9
Median age (IQR)	68 (62–85) yrs
Women (%)	88
Emphysema (%)	0
FEV1 (ml)	1694
FEV1(%)	105.4
DLCO(%)	81
Histology (%)	Adenocarcinoma (55) Squamous cell carcinoma (45)

Table 4 analyses risk by sex and age. We did not find an increased risk in any of the AATD genotypes, neither by sex nor age.

**Table 4:** Relationship between AAT genotype and risk of lung cancer, by sex and age

	AAT genotype and risk of LC among women never-smokers				AAT genotype and risk of LC among men never-smokers			
Variable	Cases, n (%)	Controls, n(%)	Adjusted OR^a^ (95% CI)	Adjusted OR^b^ (95% CI)	Cases, n (%)	Controls, n(%)	Adjusted OR^a^ (95% CI)	Adjusted OR^b^ (95% CI)
Alpha-1 antitrypsin								
MM	215 (71.4)	222 (64.5)	1 (-)	1 (-)	64 (79)	115 (71.4)	1 (-)	1 (-)
MS	65 (21.6)	98 (28.5)	0.66 (0.46—0.93)	0.66 (0.46—0.96)	15 (18.5)	39 (24.2)	0.73 (0.38—1.36)	0.67 (0.33—1.29)
MZ	9 (3)	15 (4.4)	0.78 (0.36—1.65)	0.64 (0.26—1.49)	1 (1.2)	3 (1.9)	0.85 (0.04—7.05)	0.74 (0.04—6.21)
SZ	4 (1.3)	2 (0.6)	1.97 (0.38—14.38)	1.70 (0.32—12.47)	0 (0)	2 (1.2)	-	-
SS	8 (2.7)	7 (2)	1.14 (0.41—3.17)	1.19 (0.42—3.48)	1 (1.2)	2 (1.2)	0.87 (0.04—9.37)	0.7 (0.03—7.55)
ETS exposure								
No	158 (52.5)	176 (51.2)	-	1 (-)	68 (84)	126 (78.3)	-	1 (-)
Yes	143 (47.5)	168 (48.8)	-	0.94 (0.68—1.29)	13 (16)	35 (21.7)	-	0.67 (0.32—1.36)
Radon exposure								
< 200 Bq/m^3^	171 (56.8)	225 (65.4)	-	1 (-)	49 (60.5)	90 (55.9)	-	1 (-)
> = 200 Bq/m^3^	130 (43.2)	119 (34.6)	-	1.43 (1.03—1.97)	32 (39.5)	71 (44.1)	-	0.87 (0.5—1.53)
	AAT genotype and risk of LC in persons aged < 65 years				AAT genotype and risk of LC in persons aged over 65 years			
Variable	Cases, n (%)	Controls, n (%)	Adjusted OR^a^ (95% CI)	Adjusted OR^b^ (95% CI)	Cases, n (%)	Controls, n (%)	Adjusted OR^a^ (95% CI)	Adjusted OR^b^ (95% CI)
Alpha-1 antitrypsin								
MM	99 (75.6)	155 (69.2)	1 (-)	1 (-)	180 (71.7)	182 (64.8)	1 (-)	1 (-)
MS	22 (16.8)	60 (26.8)	0.61 (0.36—1.02)	0.55 (0.31—0.95)	58 (23.1)	77 (27.4)	0.72 (0.49—1.05)	0.74 (0.49—1.1)
MZ	5 (3.8)	7 (3.1)	0.92 (0.27—2.74)	1.15 (0.33—3.76)	5 (2)	11 (3.9)	0.67 (0.26—1.66)	0.41 (0.13—1.17)
SZ	1 (0.8)	1 (0.4)	1.50 (0.06—38.35)	1.16 (0.04—30.11)	3 (1.2)	3 (1.1)	1.17 (0.21—6.49)	1 (0.18—5.53)
SS	4 (3.1)	1 (0.4)	3.26 (0.62—23.94)	6.24 (0.88—124.59)	5 (2)	8 (2.8)	0.66 (0.2—2.03)	0.63 (0.19—1.93)
ETM exposure								
No	76 (58)	134 (59.8)	-	1 (-)	150 (59.8)	168 (59.8)	-	1 (-)
Yes	55 (42)	90 (40.2)	-	0.87 (0.54—1.39)	101 (40.2)	113 (40.2)	-	0.88 (0.61—1.26)
Radon exposure								
< 200 Bq/m^3^	74 (56.5)	140 (62.5)	-	1 (-)	146 (58.2)	175 (62.3)	-	1 (-)
> = 200 Bq/m^3^	57 (43.5)	84 (37.5)	-	1.38 (0.88—2.17)	105 (41.8)	106 (37.7)	-	1.19 (0.84—1.7)

^a^Adjusted for age

^b^Adjusted for age, exposure to ETS, and residential radon

*AAT* alpha-1 antitrypsin, *ETS* environmental tobacco smoke

## Discussion

This study, which analyses the possible relationship between AATD and risk of lung cancer in never-smokers, is based on one of the largest sample sizes reported to date, in that it included 457 cases and 631 controls. No higher risk of lung cancer was found among individuals who were homozygous or heterozygous carriers of the most frequent AAT deficiency alleles (PI*S, PI*Z) as compared to carriers of the normal genotype (PI*MM). Similarly, no effect was observed for subjects by sex or age group.

These results are in contrast with those obtained in a previous study published in 2015 by Torres-Durán et al. [24], using a similar population, albeit with a smaller sample size, in which an increased risk of LC was found in homozygous S allele carriers (PI*SS) (OR: 4.64 95% CI = 1.08–19.92). This risk was higher in women (OR: 7.58: 95% CI 1.40–40.87) and subjects with exposure to ETS (OR: 12.10; 95% CI 1.18–123.77).

In the last 30 years, different studies have been conducted with the principal aim of analysing the possible increase in risk of LC associated with AATD. They have fundamentally been undertaken in the USA and Europe, and among these mention should be made of the results of Yang et al. [26], who conducted a case–control study in the USA, which observed a 70% increase in risk of LC associated with AAT deficiency genotypes, with predominance of adenocarcinoma and squamous cell carcinoma lineages. In a case–control study conducted on the Serbian population, Topic et al. [27] observed that being a carrier of an AAT deficiency genotype increased the risk of squamous cell carcinoma (OR: 4.51 IC95% = 1.66–12.29); however, this association was not found in adenocarcinoma and large cell carcinoma histology.. Recently, a systematic review was published [23], which included 6 studies with more than 4,000 patients and whose results suggest the existence of a relationship between AATD and risk of LC. This risk could be higher in individuals exposed to tobacco smoke and in those with diagnosis of COPD. This might be linked to the effects of AATD at a pulmonary level and their close relationship with tobacco use, which is the principal factor implicated in early development of emphysema in patients with severe AATD. Indeed, some studies have highlighted the association between pulmonary emphysema and risk of lung cancer [28].

Different mechanisms have been proposed which could be implicated in this association [19]. Among these, one of the most important relates to pulmonary damage caused by the decrease in plasma AAT concentrations, which gives rise to a protease-antiprotease imbalance and, in turn, favours the development of an inflammatory state that promotes carcinogenesis and tumour progression [18]. Another of the mechanisms proposed would be air trapping associated with pulmonary emphysema, since this could increase the time of contact with agents inhaled through the airway, thus increasing exposure to environmental carcinogens [29] and, by extension, to all those contained in tobacco smoke.

Analysing the possible causes that would account for the fact that the current study does not show an association between risk of LC and being a carrier of AAT deficiency alleles, when compared to the results of the previous study [24], various factors could be considered: firstly, the current study found a smaller percentage of S allele carriers than did the previous study (21.9% currently vs. 25.9%), and the same applies to Z allele carriers (3.7% currently vs. 5.2%). Likewise, the percentage of patients exposed to ETS was also smaller.

The current study also found no evidence of an increased risk of LC associated with AATD in the analysis by subgroups according to age or sex. In the previous study [24], this risk was higher among women (OR: 7.58 95% CI 1.40–40.87; *p* = 0.02), though it has to be said that the analysis was not performed in men due to the small number included (18.9%), a percentage which in the current study rose slightly, i.e., to 20.3%. To our knowledge, there is no epidemiological evidence to show that the distribution of carriers of AAT deficiency alleles in a given geographical area might be different in the two sexes. That said, however, the fact that women had a higher incidence of S allele carriers might account for these results. In contrast, the current study’s larger sample size is an indicator of greater statistical power when it comes to the validity of its results.

We analysed the characteristics of the nine cases of LC in which the PI*SS genotype was found, in view of its previously observed association with an increased risk of LC. In this sample, all but one of the cases of LC associated with a PI*SS genotype appeared in women, with 55% being adenocarcinomas and 45% being squamous cell carcinoma. Adenocarcinoma is the most frequent histological type in never-smokers, in some series attaining percentages of more than 70% [24]. Taken overall, of the cases included in this study, 77.5% were adenocarcinomas and only 7.7% corresponded to squamous cell carcinoma. Yet these relative frequencies were different in this subgroup of patients who were carriers of the PI*SS genotype, with the high proportion of squamous cell carcinoma warranting special mention, in view of the fact that these patients were never-smokers. These data agree with the results of previous studies that have analysed the relationship between AATD and LC: Topic et al. [27] identified an increased risk of squamous cell carcinoma (OR = 4.51; 95*% *CI = 1.66*–*12.29) in carriers of the PI*MZ and PI*MS genotypes; Yang et al. [30] observed an increased risk of adenocarcinoma in carriers of AAT deficiency alleles, bronchoalveolar carcinoma (OR = 2; 95*% *CI = 1.1*–*3.8) and squamous cell carcinoma in particular (OR = 2.5; 95*% *CI = 1.2*–*5.3); and Li et al. [21] also detected 55.3% of adenocarcinomas. None of these 9 patients suffered from COPD, and high-resolution computed tomography showed no presence of emphysema.

The advantages of our study are, firstly, its sample size, which is one of the largest reported to date and practically doubles the number used in our previously conducted study, including a greater number of men which thus made it possible to analyse their risk, unlike the earlier study. The exclusive use of never-smokers meant that possible biases in the interpretation of results attributable to tobacco use could be ruled out. Indeed, this and the previous study are the only ones published to date to have analysed the risk of LC associated with AATD in never-smokers. A further advantage was having access to measurements of residential radon levels, which constitute the main risk factor for LC in never-smokers and thus enabled the association between AATD and LC to be established with greater certainty.

The principal limitation of our study is common to all studies of rare diseases, in this case AATD, and relates to the low overall prevalence of the disorder, which means that the number of carriers of deficiency alleles is inevitably small irrespective of the size of sample used. Furthermore, not having plasma AAT levels means that there is the possibility that some of the genotypes rated normal by PCR might correspond to rare or null deficiency alleles, though this finding would be somewhat exceptional. Lastly, respiratory function or emphysema status was not specifically analysed in this study, something that might have been of interest in order to examine its role as an intermediary agent in lung cancer among never-smokers potentially associated with AAT deficiency.

## Conclusions

In conclusion, this study found no association between being a carrier of an AAT deficiency genotype and risk of LC in never-smokers: similarly, no association was shown in the analysis by age, sex, or exposure to ETS despite including a higher statistical power with a larger sample size, no risk association was found with the SS genotype, as was the case in our pre-study. Given that there are no other studies of these characteristics conducted on never-smokers, this could imply that AAT deficiency may only play a role in carcinogenesis of LC in smokers or ex-smokers and may have no influence in the case of never-smokers. Hence, tobacco use could act as an effect modifier in relation to the presence of this deficiency in the genesis of lung cancer. To respond to this question, other studies will have to be conducted on other populations having a different genetic base. Moreover, future research along these lines should consider exposure to residential radon, in order to be able to accurately establish the effect of AAT deficiency on never-smokers.

## Footnote

We present the following article in accordance with the STROBE reporting checklist.

## Data Availability

The datasets used and/or analysed during the current study are available from the corresponding author on reasonable request.

## Abreviations

LC: Lung cancer
AATD: Alpha-1 antitrypsin deficiency
ETS: Enviromental tobacco smoke
AAT: Alpha-1 antitrypsin
COPD: Chronic obstructive pulmonary disease
FEV1: Forced expiratory volume in the first second
DLCO: Diffusing capacity of the lung for carbon monoxide
PI: Protease inhibitor
PCR: Polymerase chain reaction
